# Experimental data on filling and emptying of a large-scale pipeline

**DOI:** 10.1038/s41597-024-03441-7

**Published:** 2024-06-07

**Authors:** Xifeng Chen, Qingzhi Hou, Janek Laanearu, Arris S. Tijsseling

**Affiliations:** 1 State Key Laboratory of Hydraulic Engineering Intelligent Construction and Operation, Tianjin University, Tianjin, 300350 China; 2 Department of Civil Engineering and Architecture, School of Engineering, Tallinn University of Technology, Tallinn, Estonia; 3 Department of Mathematics and Computer Science, Eindhoven University of Technology, Eindhoven, The Netherlands

**Keywords:** Hydrology, Environmental impact

## Abstract

Laboratory-scale experiments are one of the most important means to explore the evolution of air-water interfaces and the mechanisms of pressure oscillations in pipelines during rapid filling and emptying processes. This study presents a dataset obtained from the experimental results of the flow behaviours during the pressure-gradient-driven filling and emptying processes of a large-scale pipeline. Based on these data, it is possible to study the evolution of the water-air and air-water interfaces and their breaking during pipe filling and emptying. The experimental equipment includes a variety of components (such as tanks, valves, bends, pipes of different materials and diameters, anchors, supports and water basin) and the operation procedures are rather complex. The flow behaviours are measured by various instruments; hence a thorough hydrodynamic analysis is possible. All these features and data frameworks make the current study particularly useful as a test case for real rapid filling and emptying processes and syphoning.

## Background & Summary

Rapid pipe filling and emptying processes are common in various hydraulic applications, including water distribution networks, stormwater and sewage systems, firefighting systems, oil transport pipelines, and pipeline cleaning. During rapid pipe filling, as a high head drives the water column, air is expelled by the advancing water column. If the generated air flow is not blocked by valves, the water column expands with minimal adverse pressure and high velocity. For emptying of a pipeline initially filled with water, air is blown into the pipeline, expelling water from the system. If the driving air pressure is high and the resistance from pipe components is low, the water column will shorten, and reach high acceleration and velocity. Sudden interruptions in the advancing column during pipe filling and emptying processes lead to severe pressure changes in the system (e.g., Guo & Song^1^, Zhou *et al*.^2^, De Martino *et al*.^3^).

Rapid filling processes in hydraulics have been experimentally and theoretically studied. The laboratory tests performed by, among others, Nydal & Andreussi^4^, Liou & Hunt^5^, Zhou *et al*.^2,6^ and Vasconcelos *et al*.^7–9^ were based on relatively short pipelines with small diameters. The pipe emptying problems have garnered increasing attention in the literature. One investigated problem is bubble motion in liquids in horizontal, vertical and inclined pipes due to gravity (see e.g. Zukoski^10^, Benjamin^11^). The theoretical and experimental studies on this problem were summarized by Shosho & Ryan^12^. The focus of Zukoski^10^ was on the effect of viscosity, surface tension, inclination angle and pipe diameter on the bubble’s movement. It was found that for Reynolds numbers greater than about 200, the bubble propagation rates are substantially independent of viscous effects. Surface tension is negligible when its value is less than 10 percent of $\Delta \rho g{r}^{2}$, where *r* is the pipe radius, *g* is the gravitational acceleration and $\Delta \rho$ is the absolute value of the density difference between the primary and bubble fluid. Steady inviscid gravity currents in horizontal pipes were theoretically examined by Benjamin^11^. It was found that the flow celerity of both the bubble and the gravity current is $0.5\sqrt{gD}$, where *D* is the pipe diameter. For rapid pipe filling and emptying in hydraulics^2,4–7^, both Reynolds number and pipe diameter are generally large. Consequently, the effect of viscosity and surface tension on the bubble motion is negligible. In the large-diameter pipe the filling and emptying process result in air-water stratified flow^13,14^.

In recent years, researchers have increasingly studied the physical interaction between entrapped air and water in rapid pipeline filling and emptying. During pipe filling, rapidly compressed air pockets cause pressure oscillations that affect pipeline operation safety. Li *et al*.^15^ developed a linearized analytical model to approximate the maximum pressure and pressure oscillation characteristics due to entrapped air pressurization in a horizontal pipe when there is no significant air release. Liu *et al*.^16^ established a numerical model to investigate the maximum pressure of entrapped air pockets and its influencing factors. Aguirre-Mendoza *et al*. ^17^ developed a two-dimensional model to evaluate the hydraulic performance of the rapid filling process in a hydraulic installation with an air valve. Wang *et al*.^18^ employed a volume-of-fluid model to study pressure surges in a system with two shafts and one tunnel, verifying their findings through empty-tunnel water-filling experiments. Romero *et al*.^19^ presented a mathematical model to analyse the hydraulic transients during filling processes, validating their results with experimental measurements from the Metropolitan Area of Valencia’s water supply network. During emptying processes, air pockets expand, causing sub-atmospheric conditions, which are crucial for successful maintenance procedures. Fuertes-Miquel *et al*.^20^ developed a mathematical model to analyse the sub-atmospheric pressure pattern during the emptying process, which is important for pipe strength and air valve selection. Hurtado-Misal *et al*.^21^ used OpenFOAM software to simulate the hydraulic phenomenon during pipe emptying, analysing air pocket pressure and water velocity. Coronado-Hernandez *et al*.^22,23^ proposed an implicit formulation for computing the water column length and pressure oscillations within air pockets during the emptying process. This mathematical model underwent verification through small-scale physical model tests, showing slight enhancements compared to previous work.

It should be emphasized that the ability to perform tests and measurements in large-scale pipelines is very important. In the cases mentioned above, almost all models were validated in small laboratory facilities, with limited data available from physical systems. When filling a small-scale pipeline with relatively high driving head, the deformation of the water front shape has an insignificant effect on the overall hydrodynamics of the lengthening water column^24^. Therefore, a vertical water-air interface is often assumed to characterize the advancing flow in a horizontal pipe. However, this assumption of a plane water front is not applicable to large-scale pipelines^19^. The new large-scale tests aim to better understand the flow hydrodynamics during filling and emptying processes, focusing on the evolution of the moving interface and its impact on pressure distribution and outflow rates. Air-water flow dynamics in a rounded pipe can be reasonably well parameterized by the Froude number, but parameterization of airflow dynamics in a pipe headspace is more problematic. Therefore, Laanearu *et al*.^25^ introduced the Zukoski number, to characterize the air-cavity dynamics in the stratified-flow case of a large-scale pipeline, where the air-water interface developed during the emptying process, and in the water-filling cases as studied by Hou *et al*.^26^ and Laanearu *et al*.^25,27^.

Hou *et al*.^26^ analysed the experimental results of the two-phase flow behaviour during the rapid filling of the large-scale pipeline, and observed flow stratification and nonplanar water fronts, followed by a comparison with the results of a typical one-dimensional rigid-column mode. The simulation showed acceptable agreement with the measured values. Laanearu *et al*.^27^ used a control volume (CV) model to analyse the motion of an air-water front during the emptying of the large-scale pipeline by pressurized air. The calibrated CV model provided solutions that are qualitatively in good agreement with the experimental results. Subsequently, a new parameterization, based on the Zukoski dimensionless number, for the air–water interaction in the pipeline emptying process was proposed^25^. From these results, it was concluded that approximately 90% of the total water-column mass left the outlet during the pipeline emptying, while the remaining 10% resulted from the tail-leakage effect. Tijsseling *et al*.^28^ refined one-dimensional models for rapid filling and emptying processes with pressurized air, which can predict the flow time, flow rates and pressure distributions with acceptable accuracy.

The scale of the discussed experimental equipment is close to the actual situation in a factory; the operation procedures are complicated; the hydraulic parameters in the experimental process are measured by various instruments; and the experiments resulted in a detailed data set for the investigation of unsteady pipe flows with water-air interfaces.

## Methods

### Experimental apparatus and measuring instruments

**Fig. 1:**
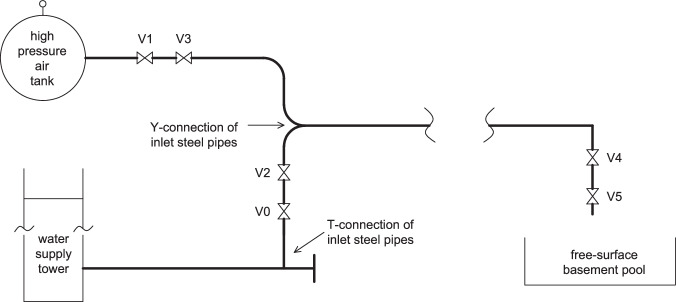
Test rig operation scheme. Valves V0 and V2 were used in the filling process: the upstream service valve V0 (DN200) was operated manually to supply water; the automatic control valve V2 (DN150) was used for flow regulation. V1, V3, V4 and V5 were used in the emptying process: the manually operated valve V1 (DN300) was used to supply air into the system; the automatically operated valve V3 (DN250) at five diameters distance from V1 was used to regulate the air flow; the downstream manual valve V4 (DN200) was used to regulate the outflow. Its orifice was maximally open at 0-degree position and fully closed at 90-degree position. The relative positions from 9/9-opening to 0/9-opening are henceforth used to characterize the outflow conditions. The manually operated on/off valve V5 (DN200), mounted three diameters downstream from V4, was used to start the emptying process. All valves were butterfly valves except valve V3, which was a cage valve.

The piping system used in the experiments is illustrated in Fig. 1. The experimental apparatus consisted of a water tank, a high-pressure air tank, steel supply pipelines (for water and air), a PVC inlet pipe, a pipe bridge, a horizontal long PVC pipeline, an outlet steel pipeline and a free-surface basement reservoir. The downstream end of the PVC bridge was defined as the origin of the coordinate system and the starting point of the test section. The *x*-coordinate follows the central axis of the pipeline, the *y*-coordinate is not used herein and the *z*-coordinate is the vertical elevation.

A water tank with a constant 25 m head relative to the centre line of the inlet was used to supply water and an air tank with a volume of 70 m^3^ was used to supply air. The water supply steel pipe, from the T-junction ($x=-\,27.2{\rm{m}}$, in practice it is a short Y-junction, but it is not important in view of the large-scale) to the upstream steel-PVC connection ($x=-\,14{\rm{m}}$), was 13.2 m long. The air supply steel pipe, from the check valve ($x=-\,43.1{\rm{m}}$) to the T-junction, was 15.9 m long. The vertical leg of the T-junction was 3.6 m long. The inner diameter of the steel pipes was 206 mm, with a wall thickness of 5.9 mm. The PVC pipe was 275.2 m long and its diameter was 250 mm with an average wall thickness of 7.3 mm. It consisted of two parts. The first part included a PVC inlet pipe and a pipe bridge. It was from the upstream steel-PVC connection to the selected starting point of the test section ($x=0\,{\rm{m}}$) and its length was 14 m. The second part was the horizontal PVC pipe of length 261.2 m (from $x=0$ m to the downstream PVC-steel connection). Most of the measurements took place in this section. The outlet steel pipe connected the downstream end of the PVC pipe to the basement reservoir. It contained two “segments” of different diameter connected by a reducer. The reducer had a length of 0.3 m and was located 0.3 m upstream of the outlet flow meter. The first segment was 8.8 m long and the diameter was 250 mm with a wall thickness of 7 mm. The second segment was 2 m long and the diameter was 200 mm with a wall thiness of 5 mm.

The PVC pipe was composed of 6 straight pipe sections (Pipe I to Pipe VI, the lengths are 39.88 m, 11.86 m, 66.59 m, 66.44 m, 12.08 m, 48.63 m). Wherever the PVC pipe needed to turn its direction, a large-radius bend ($R=5{D}_{PVC}$) was used. There were four 90-degree bends in the test section, and a long bend was used at the 180-degree turning point. The PVC pipeline was fixed to the concrete floor by metal anchors and supported with wooden blocks to reduce sagging. The 8.75 m long pipe bridge – elevated 1.3 m above the main pipeline axis – was supported by a tube-frame. However, it appeared hard to fix the most downstream elbow; at this point a very heavy mass was attached with a rope to reduce its vertical movement. The PVC pipeline segments were attached to each other by bolted connections and flanges.

**Fig. 2:**
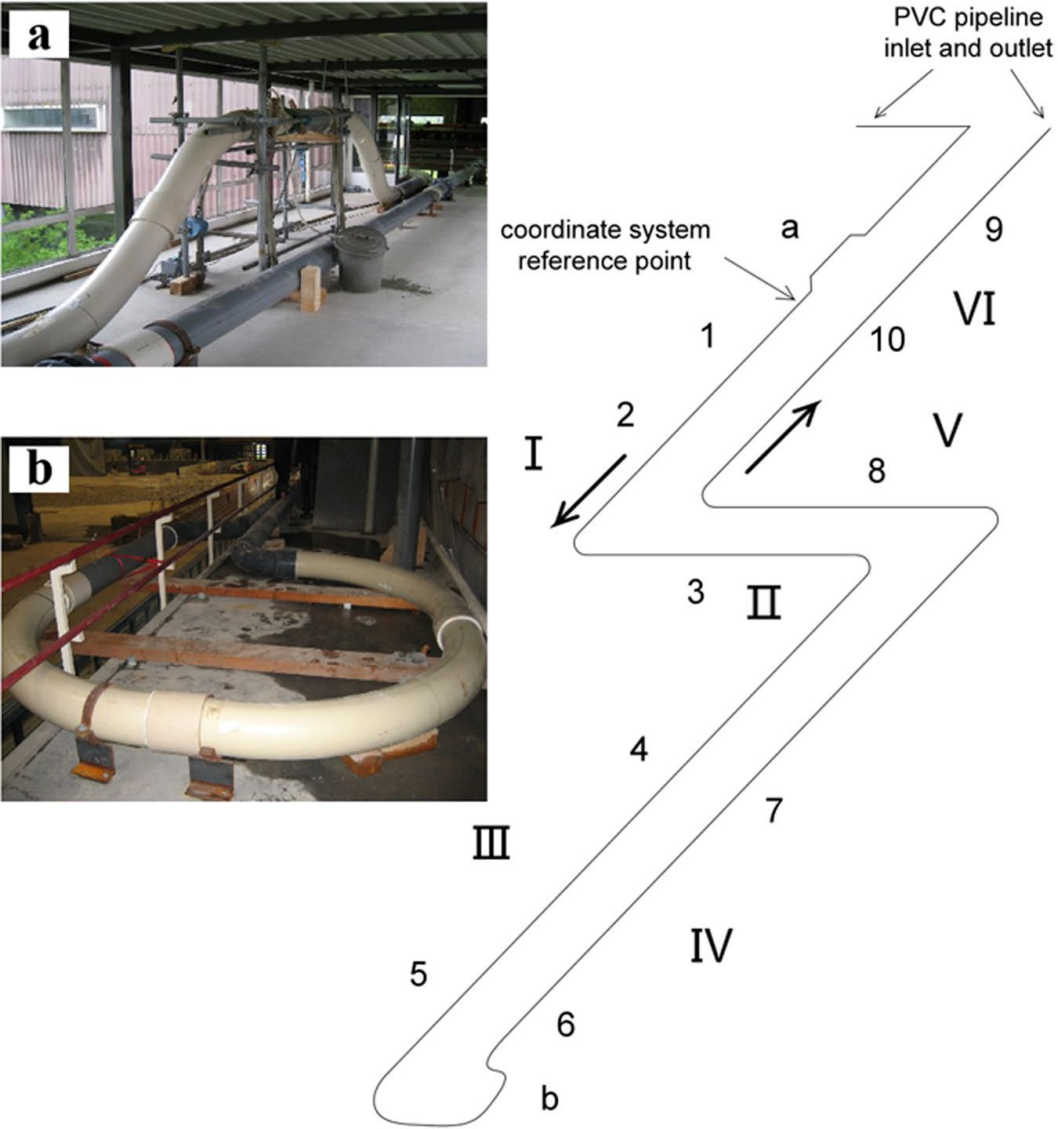
PVC pipeline layout and instrumentation; locations of measurement sections 1, 3, 5, 6, 7, 8, and 9 and transparent sections 2, 4, and 10; inset a: PVC bridge in vertical plane; inset b: 180° combined turn in horizontal plane; straight PVC pipes are indicated by roman numerals I, II, III, IV, V, and VI; pressure and water level are measured at sections 1, 3, 5, 7, 8, and 9; temperature is measured at sections 1, 3, and 9; bold arrows: water flow direction through PVC pipeline.

A maximum of 28 instruments were installed along the whole system, which is schematically and pictorially described in Fig. 2. Measurements of pressure and pipe internal water level along the PVC pipeline were taken at 6 locations and temperature at 3 locations. A removable accelerometer was used to measure pipe vibration amplitude and frequency caused by impacting liquid slugs. There were 12 pressure transducers, 6 water level meters, 4 thermometers, 3 void fraction meters and 3 flow meters. The measuring sections were numbered in sequence from upstream to downstream and the coordinates of the measuring instruments are listed in Table 1, where the type, output range, position (within pipe cross-section) and other detailed information are provided. The three transparent sections each had a length of 0.7 m, with transparent windows 0.5 m in length. A high-speed (25 fps) camera set up at these sections recorded the water-air (filling) and air-water (emptying) interface shapes and the air-water mixing process. Flow rates were recorded by two electromagnetic flow meters (EMFs) located at the horizontal inflow steel-PVC pipe connection and at the vertical outflow steel-pipe section (Fig. 1 and Table 1). The transparent Sections 2 and 10 (Fig. 2) were used to calibrate the water-level measurements with the Sony DXC-990P photo images. A sampling rate of 100 Hz was used to record the experimental quantities: inflow and outflow discharges, gauge pressures, water levels, and temperatures.

**Table 1:** DAQ channel setting, Instruments with axial coordinates, Measurement Section, Cross-Sectional Position of Pressure, Water Level, Temperature, and Flow-Rate Measurement Devices.

DAQ Channel	Output	Mark	Coordinate	Section	Range	Position	Type	Data column
—	time	—	—	—	0–200000 s	—	—	1
0	pressure	p_air_	−46.5	air inlet	0–10 bar	top	strain-gauge	2
1	temperature	T_air_	−46.3	air inlet	−100–300 °C	top	platinum resistance	3
2	flow rate	Q_air_	−47.5	air inlet	0–500 L/s	—	vortex	4
3	pressure	p_t_	—	air tank	0–10 bar	—	strain-gauge	5
4	flow rate	Q_u_	−14.3	inlet steel pipe	0–500 L/s	—	electromagnetic	6
5	pressure	p_u_	−14	inlet steel pipe	0–5 bar	left-side	strain-gauge	7
6	flow rate	Q_dv_	270.3	outlet steel pipe	0–500 L/s	—	electromagnetic	8
7	pressure	p_1_	1.6	1	0–5 bar	right-side	strain-gauge	9
8	pressure	p_3t_	46.6	3	0–5 bar	top	strain-gauge	10
9	pressure	p_3b_	46.6	3	0–5 bar	bottom	strain-gauge	11
10	pressure	p_5_	111.7	5	0–5 bar	right-side	strain-gauge	12
11	pressure	p_7_	183.7	7	0–5 bar	right-side	strain-gauge	13
12	pressure	p_8_	206.8	8	0–5 bar	right-side	strain-gauge	14
13	pressure	p_9_	252.8	9	0–5 bar	right-side	strain-gauge	15
14	water-level	WL_1_	1.7	1	12.163 mm/V	top-bottom	conductivity	16
15	water-level	WL_3_	46.4	3	12.135 mm/V	top-bottom	conductivity	17
16	water-level	WL_5_	111.7	5	12.318 mm/V	top-bottom	conductivity	18
17	water-level	WL_7_	183.7	7	11.932 mm/V	top-bottom	conductivity	19
18	water-level	WL_8_	206.8	8	12.246 mm/V	top-bottom	conductivity	20
19	water-level	WL_9_	252.9	9	12.178 mm/V	top-bottom	conductivity	21
20	temperature	T_1_	1.6	1	0–50 °C	left-side	platinum resistance	22
21	temperature	T_2_	46.4	3	0–50 °C	right-side	platinum resistance	23
22	temperature	T_3_	252.8	9	0–50 °C	left-side	platinum resistance	24
23	void fraction	VF_1_	0.5	1	0–100%	left-side	conductivity	25
24	void fraction	VF_6_	141.9	6	0–100%	right-side	conductivity	26
25	void fraction	VF_9_	251.7	9	0–100%	left-side	conductivity	27
28	pressure	p_dv_	269.5	outlet steel pipe	0–5 bar	right-side	strain-gauge	28
29	pressure	p_uv_	−27.7	inlet steel pipe	0–5 bar	right-side	strain-gauge	29

See also locations of measurement sections and straight pipes in Fig. 2; flow meter locations are shown in Fig. 1.

In this experiment, a vortex flow meter was installed in the air inlet pipeline ($x=-\,47.5{\rm{m}}$), while two electromagnetic flow meters were installed in the upstream steel pipe ($x=-\,14.3{\rm{m}}$) and downstream steel pipe ($x=270.3\,{\rm{m}}$), respectively. These three flow meters were used to measure the variation of flow rate over time. Information regarding their installation positions and measuring ranges can be found in Table 1.

The water level measurement employed a conductive water level meter, whose basic principle involves placing electrodes or contact points at different positions in the pipeline. When the water level rises to contact these electrodes, a circuit is formed, generating an electrical signal, thereby allowing the determination of the water level height to monitor its variation over time. The experimental water level measurements demonstrated excellent repeatability, indicating consistent trends in water level changes recorded by the meters across multiple repetitions.

The data from the water level meters can be used to calculate velocity, which can then be compared with the results from the flow meters to mutually validate each other. For instance, capture the moment when the front of the water flow reaches each time-marked section, and then calculate the average velocity based on the distance between the sections and the taken time. Additionally, the water level meters assist in estimating volume changes in the pipeline due to air intrusion. For example, comparing the water volume measured by the water level meters with the theoretical empty volume of the pipeline at a specific location where the water flow front reaches, the volume of air intrusion can be calculated.

### Experimental variables

All pipe filling experiments were carried out with the downstream valves V4 and V5 fully open and a constant driving head of 21.4 m ($x=34.6{\rm{m}}$, relative to $z=0{\rm{m}}$). In the pipe emptying tests, two different experimental variables were systematically investigated. The first chosen one was the upstream driving air pressure, because it highly affects the air-water interface movement. Five different air pressures of 2, 1.5, 1, 0.5 and 0 barg were used in this investigation. The last case implies that the draining is due to gravity only (driven by syphoning due to the downstream vertical pipe segment). The second experimental variable was the degree of opening of the outlet control valve V4 (0-degree – fully open; 90-degree – fully closed). By changing the closing position of valve V4 different outflow rates are obtained and the corresponding movement of the air-water interface is largely affected. Five different valve openings were tested. They were 9/9, 8/9, 6/9, 4/9 and 2/9 open. Not all the test options were combined and the thirteen test conditions used are listed in Table 2.

**Table 2:** Variables settings in the emptying experiments (valve V4 setting: 9/9 fully open, 0/9 fully closed).

Case	1	2	3	4	5	6	7	8	9	10	11	12	13
*p*_*x*_^†^	2	1.5	1	0.5	0	2	2	2	2	1	1	1	1
*ϕ*^‡^	9/9	9/9	9/9	9/9	9/9	8/9	6/9	4/9	2/9	8/9	6/9	4/9	2/9

^†^ tank air pressure (barg);

^‡^ dimensionless valve opening.

### Experimental procedure

In the pipe filling experiments, air at atmospheric pressure initially present in the system is replaced by water. Both downstream valves V4 and V5 are initially open. First, the upstream valve V0 was opened manually. Then the automatic valve V2 was opened from 0% to 15% until the height of the water in the pipe bridge reached a level of 0.4 m (0.04 barg reading from pressure transducer ${p}_{u}$ at $x=-\,14\,{\rm{m}}$). After closing valve V2 the water level gradually approached the desired height (1 m) due to a small leakage of V2. When the required water level of 1 m was reached ($x=-\,6.5{\rm{m}}$), as visually observed from a small transparent piezometric pipe, the small-size on/off valve on it was closed, and valve V2 was fully opened immediately. Then the filling process started at $t=0$. After some time (about three minutes) a steady state was reached, i.e., the inlet and outlet flow discharges were equal and constant. Then the outlet control valve V4 was closed slowly from 0 to 75 degrees to reduce possible pressure surges. After the gradual closure of valves V5 and V2 the filling process was completed.

Water entirely filling the pipeline was driven out by compressed air from the upstream high-pressure tank (pressure initially fixed between 0 barg and 2 barg). The initial conditions for each emptying run were established by the completed filling process. Air entrapped in the high-elevation air supply line (elevation 1.2 m) was ventilated through a small-size air-venting valve mounted five diameters downstream of the check valve. Similarly, air entrapped in the pipe bridge was released by a ventilating hose connected to its top. Water supply valve V0 was then manually closed, so that the unwanted leakage of valve V2 in closed position was eliminated. Then valve V4 was set to the desired degree of closing for a controlled emptying process. After valve V1 was opened (valve V3 was always open at 15% for flow regulation), the static water-column in the system was pressurized by the high-pressure air. When the induced pressure oscillations became small enough, the downstream valve V5 was opened manually as quickly as possible and then the emptying process started at $t=0$. After the main air-water front arrived at the pipe end and all water slugs were driven out of the system, valve V1 was closed and the emptying process was considered completed.

In fact, the filling and emptying experiments were continuously performed one after the other. The whole procedure included various stages: initialization for filling, pipe filling, steady-state water flow, initialization for emptying (air ventilation, valve V4 adjustment and water-column pressurization) and pipe emptying. For every test, the runs were repeated at least 5 times for nominally the same initial and boundary conditions to assess the repeatability of the unsteady two-phase flow in the pipeline and to enable statistical and error analysis.The calculated downstream valve loss coefficients have been calculated by Laanearu *et al*.^25^ (see Table 2 in ref. ^25^). Additionally, the minor loss coefficients determined through steady-state flow experiments were negligible.

### Steady-state water flow

Seventy-eight steady-state flow measurements were conducted between filling and emptying experiments, with eight dedicated to determining head losses due to surface friction and the 180-degree bend. The Darcy-Weisbach equation was used to calculate the friction factor, *f*, and minor loss coefficient, ${K}_{lb}$, at the long bend. Assuming negligible losses for the 90-degree bend due to a large radius ($R=5{D}_{PVC}$), *f* was computed using the time-averaged flow rates measured by electromagnetic flow meters upstream and downstream, along with the average pressure heads recorded at ${p}_{1}$ and ${p}_{5}$. The coefficient ${K}_{lb}$ was determined by measuring head losses between pipeline segments 5 and 7 (refer to Fig. 2). Results from the eight steady-flow experiments (see Table 3) confirmed negligible head losses attributed to the 180-degree bend.

**Table 3:** Results of steady state tests.

Run	*Re*	*f*	ε ⁄ D_PVC_	*K*_*lb*_
1	947670	0.0137	0.0001108	0.0584
2	947890	0.0137	0.0001112	0.0553
3	948710	0.0136	0.0001096	0.0533
4	947690	0.0136	0.0001086	0.0501
5	948040	0.0136	0.0001092	0.0608
6	948950	0.0136	0.0001074	0.0629
7	947720	0.0136	0.0001097	0.0691
8	950680	0.0136	0.0001044	0.0610

Subsequently, the friction factor was calculated using the measured flow rates and time-averaged pressure heads at ${p}_{1}$ and ${p}_{9}$, which were nearly identical across these eight factors, as shown in Table 3. The steady flow velocity was approximately 4 m/s, with a Reynolds number of approximately 950,000. Given the Reynolds number (*Re*) and the corresponding friction factor, the pipe relative roughness, denoted as the ratio of equivalent roughness size to pipe diameter ($\varepsilon /{D}_{PVC}$), can be computed using the Colebrook-White equation. The calculated relative roughness was approximately 0.00011.

The hydraulic grade line of the PVC pipe is linear (Fig. 3 in ref. ^26^). This is consistent with the negligible head loss calculated above due to the 180-degree long bend. The measured head at $x=-27.7$ m was 13.5 m. Initially, there is a significant head loss ($21.4-3.1-13.5=4.8$ m), primarily attributable to the valves V0 and V2 as depicted in Fig. 1. The head loss coefficient for a fully open butterfly valve is approximately ${K}_{v}=2.95$.

## Data Records

### Data availability

The dataset associated with this article is available on the repository Science Data Bank^29^ (10.57760/sciencedb.13651). The dataset is openly accessible and can be downloaded for further analysis and research purposes. Digital data will also be available from the second and third authors via personal communication.

Deltares’ 32-channel data acquisition system DAQ was used for synchronized recording of the flow rate (inflow ${Q}_{u}$, outflow ${Q}_{dv}$, air flow ${Q}_{a}$), pressure (${p}_{1},{p}_{3t},{p}_{3b},{p}_{5},{p}_{7},{p}_{8},{p}_{9}$), temperature (${T}_{1},{T}_{3},{T}_{9}$), water level ($W{L}_{1},W{L}_{3},W{L}_{5},W{L}_{7},W{L}_{9}$) and void fraction ($V{F}_{1},V{F}_{6},V{F}_{9}$). Video camera and accelerometer recordings were not electronically synchronized with the data acquisition system DAQ recordings. The notation,units and other information in the data files are shown in Table 4.

**Table 4:** Two-phase ASCII data files include RUN number in title and following columns. AVI and MP4 video files include RUN number in title.

Notation	Name	Unit	Range	Column
t	Time	ms	0–200000	1
Q inflow	Flow rate	lps	0–500	6
Q outflow	Flow rate	lps	0–500	8
Q air	Flow rate	Hz	0–500	4
p_1_	Pressure	barg	0–5	9
p_3b_	Pressure	barg	0–5	11
p_3t_	Pressure	barg	0–5	10
p_5_	Pressure	barg	0–5	12
p_7_	Pressure	barg	0–5	13
p_8_	Pressure	barg	0–5	14
p_9_	Pressure	barg	0–5	15
T_1_	Temperature	°C	0–50	22
T_3_	Temperature	°C	0–50	23
T_9_	Temperature	°C	0–50	24
WL_1_	Water level	mm	12.163 mm/V	16
WL_3_	Water level	mm	12.315 mm/V	17
WL_5_	Water level	mm	12.318 mm/V	18
WL_7_	Water level	mm	11.932 mm/V	19
WL_8_	Water level	mm	12.246 mm/V	20
WL_9_	Water level	mm	12.178 mm/V	21
VF_1_	Void fraction	%	0–100	25
VF_6_	Void fraction	%	0–100	26
VF_9_	Void fraction	%	0–100	27

### File formats

Data is provided in various formats, including CSV, Excel, and PDF. Detailed descriptions of each data file are available in the dataset’s metadata.

### Data files

The dataset contains a wide range of variables and parameters relevant to the study of large-scale pipeline systems. Key data fields include but are not limited to:

- HYIII-Delft-4 documents – reports, proposal, presentations, etc.
- HYIII-Delft-4 experiments – data matrix, procedure documents (N1 – N55)
- HYIII-Delft-4 measurements – two-phase data ASCII files
- HYIII-Delft-4 camera original recordings – video AVI files
- HYIII-Delft-4 camera reduced-size recordings – video MP4 files
- HYIII-Delft-4 gallery – photos

## Technical Validation

### Data collection and experimental setup

A series of large-scale pipeline filling and emptying experiments have been conducted to obtain relevant data. These experiments took place at Deltares, Delft, The Netherlands, as part of the EC Hydralab III project, employing the following primary equipment and instruments:

- Pipeline Model: A large-scale pipeline model was utilized, featuring diameters of 250 mm with an average wall thickness of 7.3 mm for the PVC pipe and diameters of 206 mm with a wall thickness of 5.9 mm for the steel pipes. This configuration represents key features of the actual situations found in many industrial plants.
- Flow Meter: Flow rates were recorded by two electromagnetic flow meters (EMFs) located at the horizontal inflow steel-PVC pipe connection and at the vertical outflow steel-pipe section. Uncertainty was ±1.0% in flow-rate measurements.
- Pressure Sensors: Flush-mounted strain-gauge transducers were deployed at various locations along the pipeline to capture the spatiotemporal distribution of pressure within the pipeline. Uncertainty was ±0.08% in pressure measurements.
- Water Level Meter: Conductivity probes were mounted at six locations along the PVC pipeline to record the internal water level. Measurement uncertainty in water-level recordings was 15 mm.
- Temperature: Platinum resistance sensors were mounted at three locations to record the pipe internal water temperature. Uncertainty was ±0.8 °C in temperature measurements.
- Data Logger: A sampling rate of 100 Hz was used to record the experimental quantities: inflow and outflow discharges, gauge pressures, water levels, void fraction, and temperatures.

### Data processing

Data processing involved the following steps:

Data Comparison and Consistency Checks: Data from various measurement points were compared, ensuring that the results were consistent within a reasonable range.

### Experimental verification

To validate the accuracy and reliability of our experimental data, the following methods were employed:

- Multiple Repetitions: For every test, the runs were repeated at least five times for nominally the same initial and boundary conditions to assess the repeatability of the unsteady two-phase flow in the pipeline and to enable statistical and error analysis. Results indicated minimal variation among data collected in multiple experiments. The coefficient of variance, determined for all runs was around 7%.
- Numerical Modelling: Simple modellings^25,26,28,30^ of one-dimensional rigid column models and elastic column models have been carried out to interpret the measurements. The numerical results are in good agreement with the dataset presented in this paper, ensuring that model parameters and boundary conditions matched those of the experiments.

### Data quality and uncertainty analysis

Apart from the uncertainties in the measurements, there were few uncertainties in the emptying and filling processes themselves. Quality standards for data were meticulously monitored and maintained during the experiments. Uncertainty analysis included:

- Measurement Errors: Measurement errors associated with the flow meter and pressure sensors were assessed and taken into account.
- Model Parameter Uncertainty: Sensitivity analysis was conducted to evaluate the uncertainty of model parameters and their impact on the results.

## Data Availability

The code written in Matlab 2020a for experimental data processing is stored in the database (10.57760/sciencedb.13651).

## References

[1] Guo Q, Song CCS (1990). Surging in urban storm drainage systems. J. Hydraul. Eng..

[2] Zhou F, Hicks FE, Steffler PM (2002). Transient flow in a rapidly filling horizontal pipe containing trapped air. J. Hydraul. Eng..

[3] De Martino G, Fontana N, Giugni M (2008). Transient flow caused by air expulsion through an orifice. J. Hydraul. Eng..

[4] Nydal OJ, Andreussi P (1991). Gas entrainment in a long liquid slug advancing in a near horizontal pipe. Int. J. Multiph. Flow.

[5] Liou CP, Hunt WA (1996). Filling of pipelines with undulating elevation profiles. J. Hydraul. Eng..

[6] Zhou F, Hicks FE, Steffler PM (2002). Observations of air–water interaction in a rapidly filling horizontal pipe. J. Hydraul. Eng..

[7] Vasconcelos, J. G. & Wright, S. J. *Air intrusion on pipe-filling bores in quasi-horizontal pipelines*. Department of Civil and Environmental Engineering, Report UMCEE 04-01 (2004).

[8] Vasconcelos, J. & Wright, S. Applications and limitations of single-phase models to the description of the rapid filling pipe problem. *J. Water Manag. Model*. (2005).

[9] Vasconcelos, J., Wright, S. & Guizani, M. *Experimental investigations on rapid filling of empty pipelines*. Department of Civil and Environmental Engineering, Report UMCEE-05-01 (2005).

[10] Zukoski E (1966). Influence of viscosity, surface tension, and inclination angle on motion of long bubbles in closed tubes. J. Fluid Mech..

[11] Benjamin T (1968). Gravity currents and related phenomena. J. Fluid Mech..

[12] Shosho EC, Ryan EM (2001). An experimental study of the motion of long bubbles in inclined tubes. Chem. Eng. Sci..

[13] Laanearu J. & Kaur K. Two-phase CFD modelling of air-water flow transition in a horizontal circular pipe and comparisons with experimental results. 13th International Conference on Pressure Surges, 937–948, 14-16 November 2018.

[14] Kaur K, Laanearu J, Annus I (2023). Air pocket dynamics under bridging of stratified flow during rapid filling of a horizontal pipe. J. Hydraul. Eng..

[15] Li L, Zhu DZ, Huang B (2018). Analysis of pressure transient following rapid filling of a vented horizontal pipe. Water.

[16] Liu J, Zhang J, Yu X (2018). Analytical and numerical investigation on the dynamic characteristics of entrapped air in a rapid filling pipe. J. Water Supply Res. Technol.-AQUA.

[17] Aguirre-Mendoza A.M., Oyuela S., Espinoza-Román H.G., Coronado-Hernández O.E., Fuertes-Miquel V.S., Paternina-Verona D.A. (2021). 2D CFD Modeling of rapid water filling with air valves using OpenFOAM. Water.

[18] Wang Y, Yu X, Qin H, Cheng N, Yu C (2022). Analysis of pressure surges for water filling in deep stormwater storage tunnels with entrapped air-pocket using a VOF model. AQUA - Water Infrastruct. Ecosyst. Soc..

[19] Romero G, Fuertes-Miquel VS, Coronado-Hernandez OE, Ponz-Carcelen R, Biel-Sanchis F (2020). Analysis of hydraulic transients during pipeline filling processes with air valves in large-scale installations. Urban Water J.

[20] Fuertes-Miquel VS, Coronado-Hernandez OE, Iglesias-Rey PL, Mora-Melia D (2019). Transient phenomena during the emptying process of a single pipe with water–air interaction. J. Hydraul. Res..

[21] Hurtado-Misal AD, Hernandez-Sanjuan D, Coronado-Hernandez OE, Espinoza-Roman H, Fuertes-Miquel VS (2021). Analysis of sub-atmospheric pressures during emptying of an irregular pipeline without an air valve using a 2D CFD model. Water.

[22] Coronado-Hernandez O.E., Bonilla-Correa D.M., Lovo A., Fuertes-Miquel V.S., Gatica G., Linfati R., Coronado-Hernández J.R. (2022). An implicit formulation for calculating final conditions in drainage maneuvers in pressurized water installations. Water.

[23] Coronado-Hernandez OE, Derpich I, Fuertes-Miquel VS, Coronado-Hernandez JR, Gatica G (2021). Assessment of steady and unsteady friction models in the draining processes of hydraulic installations. Water.

[24] Hou Q, Zhang LX, Tijsseling AS, Kruisbrink ACH (2012). Rapid filling of pipelines with the SPH particle method. Procedia Eng.

[25] Laanearu j (2012). Emptying of large-scale pipeline by pressurized air. J. Hydraul. Eng.

[26] Hou Q., Tijsseling A.S., Laanearu J., Annus I., Koppel T., Bergant A., van’t Westende J.M. (2014). Experimental investigation on rapid filling of a large-scale pipeline. J. Hydraul. Eng..

[27] Laanearu J, Hou Q, Annus I, Tijsseling AS (2015). Water-column mass losses during the emptying of a large-scale pipeline by pressurized air. Proc. Est. Acad. Sci..

[28] Tijsseling AS, Hou Q, Bozkuş Z, Laanearu J (2016). Improved one-dimensional models for rapid emptying and filling of pipelines. J. Press. Vessel Technol..

[29] Chen X, Hou Q, Laanearu J, Tijsseling AS (2024). Science Data Bank.

[30] Bergant, A., Hou, Q., Keramat, A. & Tijsseling, A. S. *Experimental and numerical analysis of water hammer in a large-scale PVC pipeline apparatus*. Eindhoven University of Technology, CASA-Report 11-51(2011).

